# Relationship between pigment epithelium-derived factor (PEDF) and renal function in patients with diabetic retinopathy

**Published:** 2008-05-28

**Authors:** Kayako Matsuyama, Nahoko Ogata, Masato Matsuoka, Chieko Shima, Mitsumasa Wada, Nobuo Jo, Miyo Matsumura

**Affiliations:** 1 Department of Ophthalmology, Kansai Medical University, Moriguchi, Osaka, Japan; 2 Department of Ophthalmology, Kansai Medical University, Hirakata, Osaka, Japan

## Abstract

**Purpose:**

Diabetic retinopathy and nephropathy are microvascular complications in patients with diabetes that are considered to be related. Pigment epithelium-derived factor (PEDF), a strong inhibitor of angiogenesis, is significantly elevated in the blood of diabetic patients, especially those with proliferative diabetic retinopathy (PDR). The level of PEDF in the blood, on the other hand, is reported to be low in a diabetic nephropathy. The aim of this study was to determine the relationship between PEDF and renal function in patients with diabetic retinopathy.

**Methods:**

A total of 243 type 2 diabetic patients were studied. The relationship between the diabetic retinopathy and levels of PEDF, HbA1c, blood urea nitrogen (BUN), and creatinine were evaluated.

**Results:**

The mean plasma PEDF level in patients with PDR (7.69±6.14 µg/ml; mean±standard error) was significantly higher than that of mild-to-moderate nonproliferative diabetic retinopathy (5.07±4.37 µg/ml, p=0.02). The level of BUN and creatinine increased significantly as the stage of diabetic retinopathy advanced. The plasma PEDF levels were significantly correlated with the levels of BUN and creatinine (r=0.54, p<0.0001; r=0.57, p<0.0001, respectively).

**Conclusions:**

The levels of plasma PEDF increases with advances in both diabetic retinopathy and nephropathy. Thus, increased levels of PEDF in the blood may indicate microvascular damages in diabetic patients and may be predictor of the progression of retinopathy and nephropathy.

## Introduction

Pigment epithelium-derived factor (PEDF) is a potent inhibitor of angiogenesis originally isolated from conditioned-medium of cultured human retinal pigment epithelial cells [1]. PEDF inhibits retinal endothelial cell growth, migration, and suppresses ischemia-induced neovascularization [1,2].

Diabetic retinopathy is a microvascular complication of diabetic mellitus, and is a major cause of adult blindness [3]. The level of intraocular PEDF decreases with advancing stages of diabetic retinopathy [4-6]. On the other hand, we recently found that plasma levels of PEDF are significantly higher in patients with type 2 diabetes and especially high in patients with proliferative diabetic retinopathy [7].

Diabetic nephropathy, which causes renal failure, is also a serious complication of diabetic mellitus [8]. In contrast to the report of PEDF levels in the blood of patients with diabetic retinopathy [7], Wang et al. [9] reported that the PEDF level in the kidney is down-regulated and the serum PEDF level are lower in a rat model of diabetic nephropathy.

Both retinopathy and nephropathy are common microvascular complications associated with diabetes. Previous studies have shown these conditions are strongly associated, and there have been reports that renal dysfunction is a risk factor for the development and worsening of diabetic retinopathy [10,11].

The findings in the two previous reports [7,8] are contradictory; one showed that plasma PEDF level is high in patients with diabetic retinopathy [7], while the other showed that the plasma PEDF level is low in a rat model of diabetic nephropathy [8]. The aim of this study was to determine whether the plasma level of PEDF is correlated with the renal function in patients with diabetic retinopathy.

## Methods

### Subjects

This study was conducted according to the tenets of the Declaration of Helsinki and was performed after receiving approval from the Institutional Review Committee of Kansai Medical University. An informed consent was obtained from all patients.

Blood samples were collected from 243 type 2 diabetic patients (125 men and 118 women, ages 18 to 87 years, 61.7±9.3 years; mean±standard deviation) at the Kansai Medical University Hospital.

### Examination of diabetic retinopathy

All patients with diabetic mellitus underwent a standard ophthalmic examination at the time of blood sample collection. The stage of diabetic retinopathy was determined by ophthalmoscopy and fluorescein angiography, and the patients were classified according to the severity scale of diabetic retinopathy [12]. The scale was: no apparent diabetic retinopathy (NDR), mild to moderate nonproliferative diabetic retinopathy (M-NPDR), severe nonproliferative diabetic retinopathy (S-NPDR), and proliferative diabetic retinopathy (PDR). Patients with other ocular diseases such as uveitis, glaucoma, age-related macular degeneration, retinal degeneration, and retinal vascular thrombosis were excluded. However, diabetic patients with simple cataracts were included.

### Pigment epithelium-derived factor levels in plasma

PEDF levels in plasma were measured with an ELISA Kit as per the manufacture's instructions (PEDF Sandwich ELISA Kit; Chemicon® International, Temecula, CA). Briefly, blood samples were collected in tubes containing EDTA, and platelet-poor plasma was prepared by centrifugation (3000 rpm, 20 min) and stored at -80 °C before use. Before the assay, samples were thawed on ice and urea was added to a final concentration of 8 M. After incubation on ice for 1 h, samples were diluted in assay diluent, then immediately applied to the assay plates and measured according to the manufacturer’s protocol.

### Laboratory examination

The level of HbA1c was determined by automated glycohemoglobin analyzer (HLC-723G8; TOSHO Co., Tokyo, Japan), and blood urea nitrogen (BUN) and creatinine were determined by blood autoanalyzer (AU540; Olympus, Tokyo, Japan) according to the manufacturer’s protocols.

### Statistical analysis

The results are expressed as the mean±standard error of the mean (SEM), and the data were analyzed statistically by one way analysis of variance (ANOVA) with Fisher protected least-significant difference (PSLD) test when comparing groups. The correlations between PEDF and BUN or creatinine were determined by the Pearson's product moment correlation coefficient (r). A p value <0.05 was accepted as significant.

## Results

### Plasma pigment epithelium-derived factor levels and stage of diabetic retinopathy

**Figure 1:**
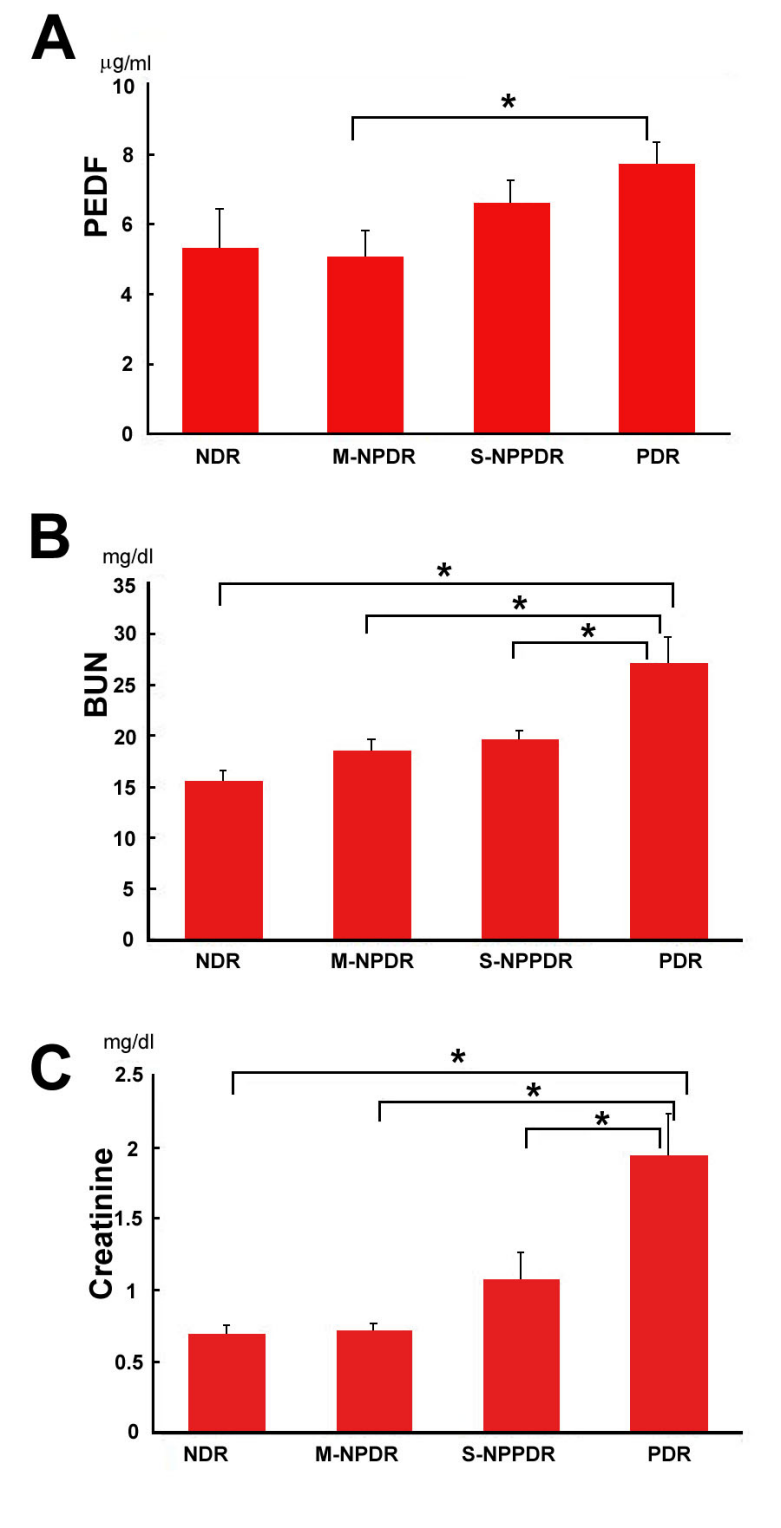
Pigment epithelium-derived factor (PEDF), blood urea nitrogen (BUN), and creatinine levels in 243 type 2 diabetic patients. **A:** PEDF levels and the stage of diabetic retinopathy. **B:** BUN levels and the stage of diabetic retinopathy. **C:** Creatinine levels and the stage of diabetic retinopathy. Abbreviations: NDR represents no apparent diabetic retinopathy, M-NPDR represents mild to moderate nonproliferative diabetic retinopathy, S-NPDR represents severe nonproliferative diabetic retinopathy, PDR represents proliferative diabetic retinopathy, and the asterisk represents a p<0.05.

The level of plasma PEDF increased as the stage of diabetic retinopathy advanced: 5.29±5.10 µg/ml in patients with NDR (n=20, 63.9±10.3 years); 5.07±4.37 µg/ml in patients with M-NPDR (n=37, 66.6±9.1 years); 6.61±6.20 µg/ml in patients with S-NPDR (n=98, 62.9±8.1 years); and 7.69±6.14 µg/ml in patients with PDR (n=88, 57.9±9.7 years) (Figure 1A). The level of PEDF in the PDR group was significantly higher than that of M-NPDR group (p=0.02).

### HbA_1_c and stage of diabetic retinopathy

The level of HbA1C was 7.2±0.3% in the NDR group, 7.4±0.2% in the M-NPDR group, 7.3±0.2% in the S-NPDR group, and 7.4±0.2% in the PDR group. The differences between the groups were not significant.

### Blood urea nitrogen, creatinine, and stage of diabetic retinopathy

The level of BUN increased as the stage of diabetic retinopathy advanced: 15.6±1.0 mg/dl in the NDR group (n=17), 18.5±1.1 mg/dl in the M-NPDR group (n=24), 19.9±0.9 mg/dl in the S-NPDR group (n=59), and 27.1±2.5 mg/dl in the PDR group (n=65) (Figure 1B). The level of BUN in the PDR group was significantly higher than that in the NDR, M-NPDR, and S-NPDR groups (p=0.002, p=0.01, p=0.003, respectively).

The level of creatinine also increased as the stage of diabetic retinopathy advanced: 0.69±0.06 mg/dl in the NDR group, 0.71±0.06 mg/dl in the M-NPDR group, 1.01±0.19 mg/dl in the S-NPDR group, and 1.94±0.30 mg/dl in the PDR group (Figure 1C). The level of creatine in the PDR group was significantly higher than that in the NDR, M-NPDR, and S-NPDR groups (p=0.01, p=0.005, p=0.008, respectively). These analyses were performed on 165 of non-selected diabetic patients who had complete sets of PEDF, BUN, creatinine, and HbA1c data.

### Correlation between pigment epithelium-derived factor level and blood urea nitrogen and creatinine levels

**Figure 2:**
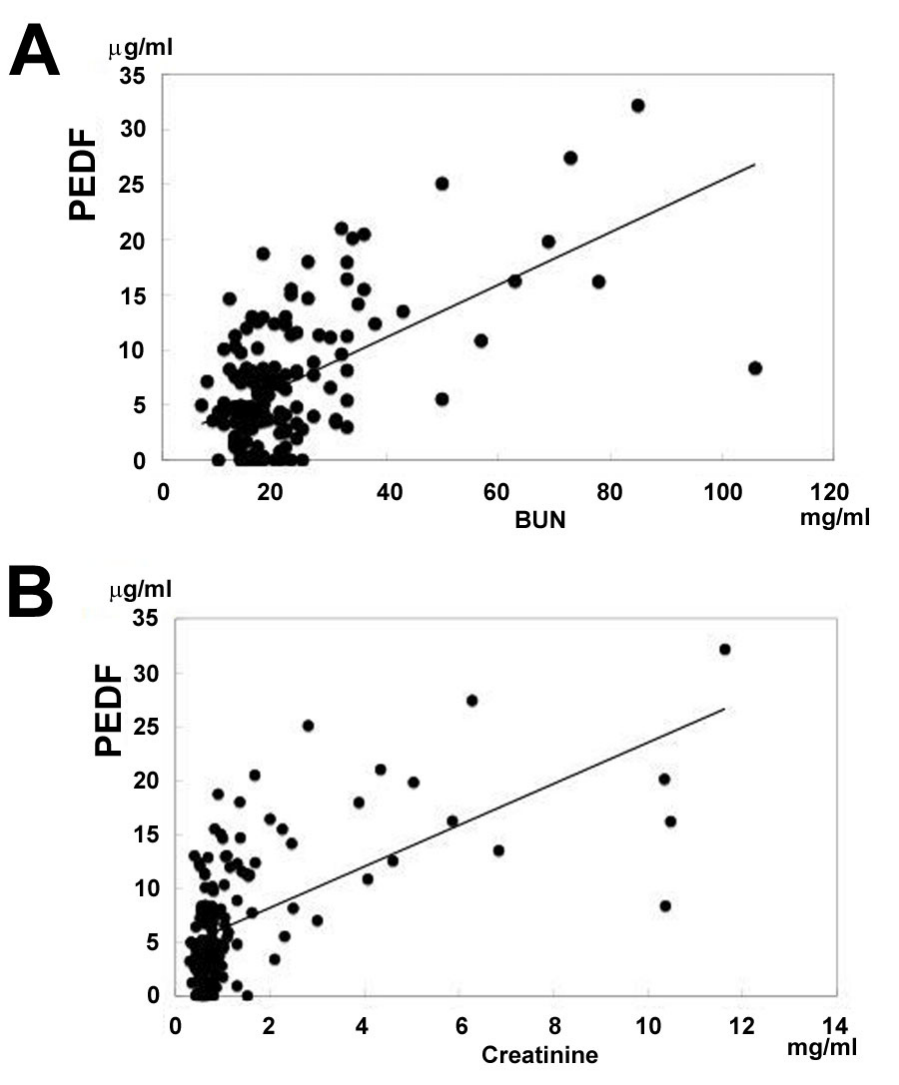
Relationship between pigment epithelium-derived factor and renal function. **A:** Correlation between pigment epithelium-derived factor (PEDF) levels and blood urea nitrogen shows a correlation coefficient of r=0.54 at a significance level of p<0.0001. **B:** Correlation between PEDF levels and creatinine shows a correlation coefficient of r=0.57 at a significance level of p<0.0001.

The plasma PEDF levels increased with increasing BUN and creatinine levels (Figure 2, n=165). The correlation between PEDF and BUN was significant (r=0.54, p<0.0001), and the correlation between PEDF and creatinine was also significant (r=0.57, p<0.0001).

## Discussion

PEDF is synthesized in a wide range of human tissues including the lung, brain, kidney, adipose tissues, and especially in the liver [1,13]. The multiple sites may be the cause of the high levels of PEDF in the blood [13].

We recently reported that plasma PEDF level in diabetic patients was significantly higher than that in controls, and the level was especially high in patients with proliferative diabetic retinopathy [7]. The results of this study confirmed those findings.

Diabetic retinopathy and nephropathy are both microvascular complications associated with diabetes. The results of several studies that have examined the relationship between diabetic retinopathy and nephropathy are not consistent [14]. We clearly demonstrated that levels of BUN and creatinine increased significantly as the stage of diabetic retinopathy advanced. These results support previous studies that showed that these conditions are strongly associated [10,11]. In addition, plasma PEDF levels were significantly correlated with the levels of both BUN and creatinine.

Several growth factors, most importantly, transforming growth factor-β (TGF-β), vascular endothelial growth factor (VEGF), and fibroblast growth factor (FGF), have been suggested to be involved in the pathogenesis of diabetic nephropathy and retinopathy [15-19].

PEDF inhibits the migration of endothelial cells induced by VEGF and FGF [1,2]. In addition, PEDF has been suggested to be an anti-inflammatory cytokine [20]. PEDF inhibits the expression of tumor necrosis factor-α (TNF-α), VEGF, monocyte chemoattractant factor-1 (MCP-1), and intercellular adhesion molecule-1 (ICAM-1) [20]. PEDF also significantly inhibits the activity of advanced glycation end products (AGEs) in microvascular endothelial cells [21]. From these observations, PEDF has been suggested to play a protective role against vascular damages by suppressing proliferative inflammatory responses to injuries in endothelial cells.

Chronic, low-grade inflammation is responsible for diabetic microangiopathy, such as retinopathy [22] and nephropathy [23]. Thus, the increase in the levels of PEDF in the plasma of diabetic patients with diabetic retinopathy and nephropathy may be a counteractive system that inhibits the vascular damages in diabetic mellitus. Yamagishi et al. [13] also reported that serum PEDF levels may increase as a counteractive system against coronary risk factors in the metabolic syndrome. Together with the previous report, PEDF is most likely associated with metabolism and may be associated with angiopathy.

In contrast to our results, an earlier study demonstrated that the expression of PEDF was decreased at both the mRNA and protein states in the kidney of diabetic rats induced by streptozotocin. The PEDF levels in the serum in these rats were also significantly decreased at the late stage of diabetes when compared with the age-matched non-diabetic control animals [9]. We still do not know the exact reason for this discrepancy; however one reason may be that they used an animal model of type 1 diabetes induced by streptozotocin, whereas we examined humans with type 2 diabetes.

Although we found that the levels of plasma PEDF increased with advances in both diabetic retinopathy and nephropathy, the level of intraocular PEDF has been shown to decrease with advancing stages of diabetic retinopathy [4-6]. Because, PEDF is a potent anti-angiogenic and anti-inflammatory cytokine [1,2,20,21], PEDF may be consumed in the eye with diabetic retinopathy to counteract the angiogenic and inflammatory responses of the endothelial cell, which would then lead to lower levels. A similar mechanism may apply to diabetic nephropathy and may account for the decreased expression of PEDF in the kidney of diabetic rats [9].

A more recent study found high levels of PEDF in the serum of patients with end-stage renal disease [24] which is compatible with our results. Because of the close relationship between diabetic nephropathy and diabetic retinopathy and because both of these conditions result in diabetic microangiopathy, it is more likely that PEDF is associated with the metabolism of diabetic patients and may be associated with the angiopathy.

Estimates of the prevalence of retinopathy are usually based on direct examination of the anatomic retinal changes, whereas those of nephropathy are defined by functional abnormalities such as microalbuminuria or overt proteinuria. Further longitudinal studies with repetitive samples from the same donor should be performed to determine the changes with time. These findings should answer the question on whether the increased levels of PEDF in the blood of patients with diabetic retinopathy would indicate microvascular damage and may be used to predict the progression of retinopathy and nephropathy.
